# Defining System Requirements for Simplified Blood Culture to Enable Widespread Use in Resource-Limited Settings

**DOI:** 10.3390/diagnostics9010010

**Published:** 2019-01-11

**Authors:** Peter J. Dailey, Jennifer Osborn, Elizabeth A. Ashley, Ellen Jo Baron, David A. B. Dance, Daniela Fusco, Caterina Fanello, Yukari C. Manabe, Margaret Mokomane, Paul N. Newton, Belay Tessema, Chris Isaacs, Sabine Dittrich

**Affiliations:** 1 Foundation for Innovative New Diagnostics (FIND), Malaria and Fever Program, Chemine des Mines 9, Geneva 1202, Switzerland; Peter.Dailey@finddx.org (P.J.D.); jennifer.osborn@finddx.org (J.O.); chris.isaacs@connected-dx.com (C.I.); 2 Division of Infectious Disease & Vaccinology, School of Public Health, University of California, Berkeley 94720, CA, USA; 3 Centre for Tropical Medicine and Global Health, Nuffield Department of Medicine, University of Oxford, Oxford OX1 3SY, UK; liz@tropmedres.ac (E.A.A.); david.d@tropmedres.ac (D.A.B.D.); Caterina@tropmedres.ac (C.F.); Paul.Newton@tropmedres.ac (P.N.N.); 4 Myanmar Oxford Clinical Research Unit, Yangon, Myanmar; 5 Department of Pathology, Stanford University, Palo Alto 94304, CA, USA; ejbaron@stanford.edu; 6 Lao-Oxford-Mahosot Hospital-Wellcome Trust Research Unit, Microbiology Laboratory, Mahosot Hospital, Vientiane, Lao PDR; 7 Faculty of Infectious and Tropical Diseases, London School of Hygiene and Tropical Medicine, London WC1E 7HT, UK; 8 Pole of Clinical Research, French Institute of Health and Medical Research, Paris 75013, France; fusco@bnitm.de; 9 Department of Epidemiology of Infectious Diseases, Bernhard Nocht Institute for Tropical Medicine, Hamburg 20359, Germany; 10 Kinshasa Mahidol Oxford Research Unit, University of Oxford and Kinshasa School of Public Health, Kinshasa, The Democratic Republic of Congo; 11 Division of Infectious Diseases, Department of Medicine, School of Medicine, Johns Hopkins School of Medicine, Baltimore, MD 21205, USA; ymanabe@jhmi.edu; 12 National Health Laboratory, Gaborone, Botswana; bafanamargaret@gmail.com; 13 Department of Medical Microbiology, College of Medicine and Health Sciences, University of Gondar, Gondar, Ethiopia; bt1488@yahoo.com

**Keywords:** blood culture, hemoculture, target product profile, low- and middle-income countries, LMICs, resource limited, microbiology, AMR, sepsis

## Abstract

Bacterial blood stream infections (BSI) are a common cause of mortality and morbidity globally. As the causative agents and the resulting treatment decisions vary, near-patient testing and surveillance tools are necessary to monitor bacterial causes and resistance to antimicrobial agents. The gold standard to identify BSIs is blood culture (BC), a methodology not widely available in resource-limited settings. The aim of the study was to map out a target product profile of a simplified BC system (SBCS) to inform product development efforts. To identify the desired characteristics of a SBCS, we enlisted a small group of specialists working in Africa and Asia. Questions were used to understand challenges and how these constraints inform system requirements. The specialists were infectious disease physicians, public health/clinical microbiologists, clinical researchers, and technology experts with different geographical backgrounds. All suggested that BC should ideally be available at the district hospital level. Many of the same operational challenges, such as limited availability of culture bottles, electricity and internet connectivity, profuse dust, the lack of ambient temperature control, and human capacity constraints were identified across the different regions. BCs, although the accepted gold standard for diagnosis of BSIs, are not widely available outside of reference/research centers in Africa and Asia. To extend the reach of this important tool, it is crucial to engage product developers and academic research partners to develop accessible alternatives.

## 1. Introduction

The World Health Assembly and World Health Organization (WHO) have recognized sepsis as a global public health priority [1]. Sepsis is a major worldwide health and economic burden [2] and it has been estimated that there are 31 million cases of sepsis per year worldwide with 6 million patient deaths [1]. Sepsis is also a frequent cause of the death of neonates and children less than 5 years of age [3]. Blood culture is an essential laboratory procedure that can influence the treatment of patients with sepsis [4]. Results of blood cultures (BCs) also play a critical role in informing regional empiric therapy guidelines and the tracking of antimicrobial susceptibility trends and patterns [2]. BC was pioneered in the early and mid-20th century [5] with minor later improvements in the method due to the automated detection of positive BCs [6]. In recent years, new technologies were developed to enable more rapid identification of pathogens and detection of resistance markers from positive BCs [7]. However, they have not replaced the basic elements of conventional BC or the testing that is required following the detection of a positive sample, like identification (ID) of the organism and antimicrobial susceptibility testing (AST). To this day, BC remains the gold standard method to detect bloodstream infections and for tracking of antimicrobial resistance (AMR), despite being labor intensive and complex [4]. Unfortunately, clinical microbiology laboratory capacity is limited and underutilized in many low- and middle-income countries (LMIC) [8,9,10,11,12], a shortcoming increasingly recognized in the context of AMR interventions and surveillance. BCs are challenging to implement due to the length of time to return results to patients and the perceived lack of impact on treatment. Further, there is a necessity for sophisticated laboratory facilities and high costs due to the need for a multitude of different reagents. Further, highly-trained staff is also required at all steps, requiring sophisticated sustainable capacity implementation. Uptake by clinicians may also be poor due to the length of time from sample collection to result and inadequate liaison between the laboratory and wards and the often-perceived lack of quality and utility of the results from the laboratory [8,10,13]. Recent international initiatives have been directed at improving laboratory capabilities in low-resource areas with a specific focus on general microbiology facilities [14,15]. Along with the extensive capacity improvement activities that are ongoing to support the global fight against rising AMR, there is a need to enrich the diagnostic landscape with new tools appropriate for use in these regions [16]. The purpose of this work was to draft a target product profile (TPP) to inform product developers of the requirements of a simplified BC system applicable in resource-limited settings. TPPs inform academic and commercial product developers of key characteristics and the performance specifications of a test system that are required to meet the end users’ needs for a defined use case [17,18,19]. Beyond informing technology innovators, the development of TPPs helps to raise the profile and promote the need for a certain type of diagnostic tool. TPPs include optimal and minimal definitions for each test performance characteristic. Optimal characteristics describe the ideal, but often technically challenging, specifications. The minimal requirements define the user needs essential to meet the intended use of the test system to be a useful diagnostic tool. Ideally, products should be designed to achieve as many of the optimal characteristics as feasible, while still satisfying the minimal criteria for all defined features.

We sought to define product features that address challenges specific to resource-limited settings and encourage product development efforts for simplified BC systems that are suitable for these localities and could be used as near-patient tests hospitals outside of the big reference centers.

## 2. Materials and Methods

### 2.1. Specialist Interviews

A series of eight interviews (nine individuals at eight separate locations) were conducted to explore the needs and barriers for performing BCs in resource limited settings (authors: E.A.A., E.J.B., D.A.B.D., D.F., Y.C.M., M.M., P.N.N., and B.T.). Stakeholders were a convenient sample of experts with relevant and regional expertise. Stakeholders included infectious disease physicians (*n* = 3), public health/clinical microbiologists (*n* = 4), a clinical researcher (*n* = 1), and a technology expert (*n* = 1). They were based at hospitals in Asia (*n* = 3) and Africa (*n* = 5), or in the USA (and multiple low-resource areas = 1). Stakeholders were selected to represent laboratory settings ranging from level 4 to level 2 health facilities [20], with or without ongoing BC testing capacity. Interviewees were selected to represent experiences from different regions and levels of capacity to establish needs and gaps across a spectrum of laboratories, settings and cultures. All were affiliated with public sector sites and/or academic institutions. Structured phone interviews (~60–120 min) were conducted using an interview guide that included an overview of the workflow of standard BCs to guide the discussion. Specific questions were developed by the FIND-team (authors: P.J.D., J.O., and S.D.), which addressed each BC process step from sample collection to results to understand current procedures, needs, and challenges (Supplementary Materials). The same guidance documents were used for all calls and the same interviewers were in the calls to avoid bias. The aim was to summarizes a broad range of topics that could which were then used as the basis of a more simplified blood culture system, assuming that the current system was suboptimal. Responses were summarized in an Excel database and, after obtaining oral or consent, all interviews were voice recorded for later verification. Commonalities between respondents were summarized and key findings were distilled from these records.

### 2.2. Target Product Profile Development Process

A list of the essential product characteristics for a simplified BC system was compiled based on FIND internal templates (https://www.finddx.org/target-product-profiles/). These key characteristics and the responses from the interviews were used to develop an initial TPP draft. Published standards and guidelines for conventional BC system requirements were also incorporated into some of the minimal and optimal characteristics, where applicable [20,21]. To obtain the TPP presented here, an adapted Delphi-like process [18] was used. In brief, the first draft of the TPP was developed from the interview responses and shared with all specialists, then comments and corrections were collected and incorporated for the next version. The second draft of the TPP was shared back and agreement was thought for more controversial characteristics in individual communication. Previous published definitions [22] for the health system levels were used in the TPP and “near-patient testing” in this case does include laboratory testing, albeit outside of big reference centers.

## 3. Results

### 3.1. Current Use of BCs in Low Resource Settings

Only half (4/8, 50%) of the interviewees worked at sites currently operating a BC system (Table 1). Sites were donor-supported and not typical of most hospitals in their countries. The remaining stakeholders (*n* = 4) had in-depth knowledge of settings or facilities representative of their region, though their current sites were not actively performing BC. A multitude of challenges were described by all (Table 2) and these challenges were then used to inform the TPP characteristics outlined below. Of the four sites performing BCs, only one was open 24/7, and this was a reference level health facility. Three of four sites performed Gram stains on positive BCs. Reliable patient identification was raised as a point of concern in multiple settings as many patients do not know their birthdate, names are often very similar, and many countries do not have national identification numbers or effective hospital patient numbering systems. All four BC sites were collecting blood from patients by syringe and needle following skin decontamination with alcohol and/or iodine. Only (2/4, 50%) had access to “butterfly” needles for difficult and pediatric draws. Their use was impeded by the additional cost. Failure to collect the recommended blood volume was commonplace (7/8, 88%), despite standard guidelines for BC in adults recommending drawing 10 mL of blood in two bottles (total 20 mL), two to three times [21]. In many settings described in the interviews, this is not common practice and usually only one bottle is collected. In some locations, particularly in Southeast Asia interviewees described a general reluctance to draw large volumes of blood. The information collected in the interviews was used to develop this preliminary TPP (Table 3). Describing the challenges and translating the current shortcomings into technical requirements in the TPP characteristics aims to inform researchers and provide the basis for transformative innovation.

### 3.2. Target Product Profile

#### 3.2.1. Scope of the test

Stakeholders emphasized the importance of BCs in providing data for pathogen and AMR surveillance and in improving local empiric treatment guidelines. Based on the stakeholders’ input, the target population was defined as: All patients presenting with fever, including neonates and immunocompromised individuals. The acceptable target user was defined as a moderately trained technician, with 3–6 months of training. This was specified because several (3/8, 38%) respondents noted that staff retention was a significant barrier. Stakeholders agreed that BC systems should be available at level 2 facilities. For all but one site (Lao PDR) in which a long-term research project supported BCs, patients were required to pay for BCs. The acceptable per test cost was determined to be < $10 USD, with < $5 USD desired if BCs are predominantly used to inform patient care.

#### 3.2.2. Test Performance

Interviewees agreed that a simplified system should include automated detection of a positive culture and ideally be able to identify pathogens and perform AST. The diagnostic accuracy values were estimated and set to > 95% sensitivity for detection of positive BC using standard blood culture methodologies, for either a mono-microbial or a poly-microbial infection. The ability to distinguish poly- from mono-microbial infection was considered an optimal requirement. TPP requirements for interfering substances were specifically drafted to address conditions common to LMICs, such as the frequent use of over the counter antibiotics [27] and malaria coinfection.

#### 3.2.3. Test Procedure

All interviewees reported a preference for systems that would reduce staff errors and training requirements and increase clinician trust in BC results. The TPP characteristics were informed by the level of staff training and test complexity typical to level two facilities based on predefined criteria for microscopy centers [19]. Blood sample collection was consistently reported to be a challenge and stakeholders reported inadequate training and complex blood collection procedures resulting in contamination rates as high as 15% (reported range: 5–10%), compared to 3–5% in the USA or Europe and an international target of 2–3% [22,32]. To address cases where significant sample transport time or delayed entry into an automated system may occur, incubation of culture bottles at 35 °C was preferred. All sites reported inadequate or non-existent quality control of BC bottles or media. For sites that prepared their own media, half reported minimal checks for sterility of the media preparation. In one country, media is purchased by many laboratories from one centralized manufacturer, which does extensive quality control on culture media.

#### 3.2.4. Test Results

To inform the TPP characteristics related to test results, we discussed how BC results were reported in each facility (Table 1). Results were not available in a timely fashion because the laboratory did not often communicate preliminary results at six of eight sites (75%). As a minimum requirement, reporting of preliminary results of a positive BC, ideally with Gram stain information, is required to inform clinical decisions as quickly as possible. For the minimal requirements, results from antimicrobial susceptibility testing by a separate method were acceptable. However, interviewees unanimously expressed an interest in automated AST information from an integrated simplified BC system, which was included as the desired characteristic. The addition of therapy recommendations based on local treatment guidelines to accompany BC test results was mentioned as a useful addition to facilitate treatment guidance for clinicians by 3/8 (38%) interviewees and was included as an optimal requirement for data interpretation and output.

#### 3.2.5. Consumables and Operational Characteristics

The optimal requirement calls for inclusion of sample collection supplies with the BC bottle test kit to address this limitation. Media formulations can have significant impact on a technology’s detection method, hence, the minimal requirement was restricted to compatibility of a designated manufacturer’s BC bottle and the optimal case would allow for locally produced media formulations. Several sites expressed an interest in biosafety alerts for hazardous agents including *Burkholderia pseudomallei*, *Brucella* spp., and *Bacillus anthracis.*

#### 3.2.6. Cultural Considerations Beyond the TPP

A recurring theme brought up by the specialists was that technology solutions alone will not address all the challenges currently impeding the use of BCs in resource-limited settings. Even if a timely result was provided to clinicians, stakeholders reported that a lack of trust in results often leads clinicians to ignore laboratory results in their decision-making. Stakeholders suggested that clinicians need education on the use of empiric therapy guidelines and BCs, since limited availability of quality BC facilities can result in a lack of knowledge and training. There is a need to educate clinicians to drive demand for BC from the clinician, not from the laboratory, and regularly enhanced laboratory-ward/outpatient clinical liaison. Further, supply chain issues, stock-outs, and bureaucratic challenges are barriers for diagnostic and therapeutic availability. Based on the small sample number, it appeared that supply chain issues were more prevalent in Asia.

## 4. Discussion

Faster and more accurate diagnosis of bloodstream infections in low-resource areas is critical for effective patient management, surveillance of AMR, and development of local empiric therapy guidelines. For example, based on the interview feedback Guinea, a county of 12.4 million inhabitants, does not have a microbiology facility capable of performing pathogen ID and AST, even at the highest level of the health system. This results in a lack of data to inform treatment and public health interventions [11]. In parallel to the work reported here, the BACTI-LRS consortium also highlighted this need in a similar initiative, which explored requirements to implement bacteriology in resource-limited setting LMICs [8].

The aim of the current report was to identify the challenges and gaps to performing BCs and suggest how new innovations could address them in the form of a simplified BC system. Although this is one solution to the problem, the general lack of laboratory capacity, supply chain constrains, and human and infrastructure hurdles remain, and need to be addressed in parallel. Albeit anecdotal, interviewees hinted at overall improved supply chains following the U.S. President’s Emergency Plan for AIDS Relief (PEPFAR) initiative that also resulted in better microbiology supply access. This is encouraging and demonstrates that barriers can be overcome if global health players unite around the same goals. In addition to the technical and capacity challenges reported by the interviewees, our findings highlight the cultural issues and the need for continued education around the use, utility and safety of BCs.

Although this report adds new knowledge on the technical needs, the work also has limitations that might have introduced bias and might justify additional work and consensus building if this work results in significant interest by product development partners. In brief, our interviews included only a small number of stakeholders representing two continents who might have given a narrow view of the situation. Further, the interview team assumed that current systems are suboptimal, potentially further introducing bias. Although this is possible, recent publications have identified the lack of quality data on AMR and large international initiatives (e.g., Fleming Fund, UK) are underway to bridge the challenges reported here [8,10,11,33,34]. This suggests that problems highlighted in our small sample set are to a large degree transferable beyond the ’interviewees’ countries and setting. In addition, the TPP might have been influenced by preconceived notions of what a simplified BC tool should look like. However, given the separation of minimum and optimal criteria, as well as an extensive list of 33 characteristics, it is believed that the TPP will be widely applicable and lessons learned here can be applied to BC systems.

The vision for the TPP is to simplify work for the laboratory needs, while not necessarily being simple for the innovator. In general, despite the daunting list of requirements, it should be noted that a system that meets a subset of the characteristics and is designed with the procurement and capacity challenges of resource-limited settings in mind would be a substantial improvement on current BC systems.

In conclusion, a simplified BC system would enable expanded use of BCs in low-resource areas. BC is an essential tool to inform treatment and management of bloodstream infection and the interviews with different stakeholders highlighted the continued need for phenotypic detection tools beyond diagnostic tools that can only identify selected pathogens and resistance markers. Tools like these provide crucial surveillance information that is currently largely unavailable in LIMCs [11]. To ensure that all these tools are available, a joint effort from academia and industry, as well as global health organizations, is needed to seed the pipeline of innovations and ensure that infrastructures at all levels of the health care system are in place to accept improved BC technology. In an ideal scenario, these improved capacities extend beyond the reference level and cover all levels of the health system to ensure all severely sick patients in need of appropriate treatment at hospitals can receive a diagnostic test that will inform the choice of antibiotics for improved outcomes, as well as fostering antibiotic stewardship and AMR surveillance.

## Figures and Tables

**Table 1:** Overview of blood culture (BC) methods in use at interviewed sites or representative countries. Only aerobic cultures were performed across all sites, and bottles were incubated for seven days. Costs at interview sites ranged from a cost of $5–7 USD per test and up to $25 USD was reported in one case. All sites currently performing BCs reported that nurses were the primary health providers collecting blood samples, though at some sites physicians (2/4, 50%) and lab technicians (1/4, 25%) also collecting samples. The expertise of the interviewees from the different countries were infectious disease physicians (Lao PDR, Myanmar, Cambodia), public health/clinical microbiologist (Ethiopia, Botswana).

City, County	Level [22]	Current System	Media Preparation	Delayed Entry	QC of Reagents	Reporting Protocol	Identification	AST
Vientiane, Lao PDR	3–4	Manual, daily check for turbidity; blood	Local	Sample transport from remote locations, try to receive within 24 hours of collection; transport at RT in a double-skinned insulated metal box	QC for agar made in-house	Gram stain, some rapid dx tests performed and the preliminary result is communicated verbally to clinicians ASAP and updated on a daily basis until there is a written a final report	API^®^ (bioMerieux)	Disk diffusion
Multiple, Cambodia	3	Manual, daily check for turbidity	Local	Ideally immediate; transport at RT	Central QC for media at manufacturer	Lab to call ward to inform doctor of result	Manual biochemical	Disk diffusion
Gondar, Ethiopia	3–4	Manual, daily check for turbidity	Local	Ideally immediate, can be small delays; transport at RT	QC on media for sterility	Preliminary reports: growth (turbidity)—report Gram stain preliminary report, and finally drug susceptibility	Gram stain and then biochemical tests for Gram negative organism	Disk diffusion
Gaborone, Botswana	4	Manual, daily check for turbidity	Procure	Ideally immediate, though delays up to 8 h; transport at RT	QC on media	Positive results reported in Laboratory Information Systems for some facilities; but manual reports are collected by hospital wards daily	Manual biochemical	Disk diffusion
Multiple, Myanmar	3–4	Majority are manual	Local	Overnight delays > 12 h are common; transport at RT	No	Final report (~7 days)	Manual Biochemical (few level 4 centers are introducing Vitek2)	Disk diffusion (automated methods available for selected patients at very few Level 4 centers)

ASAP: As soon as possible; dx: Diagnosis; QC: Quality control; RT: Room temperature; AST: Antimicrobial susceptibility testing.

**Table 2:** Summary of challenges for performing BC in low-resource settings, based on responses from interviewed specialists in multiple countries. Responses were subjective assessments by the interviewed and are not necessarily transferrable to all settings in the respective country. The following definitions for three colors were used: Green = no challenge, yellow = some challenges, red = significant challenges, white = unsure/question not answered. The expertise of the interviewees from the different countries were infectious disease physicians (Lao PDR, Myanmar, Cambodia), public health/clinical microbiologist (Ethiopia, Botswana, Guinea) and clinical researcher (DR Congo).

		**Environmental Challenges**						**Implementation Challenges**				
Country	Available at level	Power	Dust	Lab Temperature control	Humidity	Connectivity		Level of lab staff training	Quality Control for BC	Sample Collection (Volume, training)	Specimen Tracking	Result Reporting

Lao PDR	3–4											
Cambodia	3											
Ethiopia	3–4											
Botswana	4											
Guinea	4 *											
Myanmar	3											
DR Congo	3											
Uganda	3–4											

* Only urine culture capacity available at the capital. PDR: People’s Democratic Republic, DR: Democratic Republic.

**Table 3:** Proposed target product profile for a simplified BC system based on stakeholder input and international guidelines.

Characteristic	Minimal	Optimal	Additional References
**SCOPE**			
**Goal**	A simplified blood culture system suitable for resource limited settings to support patient management and surveillance activities		
**Target population**	Total population (including neonates and immunocompromised individuals) presenting with fever		
**Target level of health system**	Level 3 (Regional/Provincial Hospital) and above	Appropriate for use in level 2 (District Hospital)	[22]
**Target user**	Moderately trained lab technicians (e.g., 1–2 year certificates)	Lab technicians with limited training (e.g., 3–6 months, able to operate an integrated test with minimal additional steps)	[9]
**Platform cost**	< US $20,000	< US $5000	
**Price of individual test (2 culture bottles)**	< US $10 per test	< US $5 per test	
**TEST PERFORMANCE**			
**System detection capabilities**	- Culture positivity, Gram status; - Antimicrobial susceptibility can be determined with additional methodologies	Pathogen identification and antimicrobial susceptibility are automated outputs of the system	
**Pathogen detection**	> 95% sensitivity for detection of positive BC for either monomicrobial or polymicrobial		
**Pathogen identification**	Identifies 90% of isolates to species level, 95% genus level	Identifies 95% to species level, 99% to genus level	Standard BC and identification by MALDI-TOF MS is the reference [23,24].
**Ability to determine the presence of mixed BCs**	Not able to determine monomicrobial from polymicrobial infections	Able to determine monomicrobial from polymicrobial infections	
**Interfering substances**	Able to provide an accurate result in the presence of malaria infection [25,26]	Able to provide an accurate result in the presence of malaria infection and/or antibiotics [27]	
**TEST PROCEDURE**			
**Ease of use/test complexity**	The entire test procedure for system operation after sample collection to result should require a maximum of 2 steps by the user	The entire test procedure for system operation after sample collection to result should require a maximum of 1 step by the user and no additional steps required by user after the sample has been placed into the instrument	[19]
**Sample volume**	- Test consumable (culture bottle) should support smaller volumes (5 mL or less) for pediatric samples and low volume draws; - Separate culture bottles for pediatric samples are acceptable	The same test consumable (culture bottle) should support smaller volumes (5 mL or less) for pediatric samples and low volume draws	International and local guidelines regarding blood volumes and number of samples collected should be followed
**Delayed entry**	- Allows for room temperature storage of BC bottles post collection for <4 h prior to culture; - If sample transport takes longer than 2 h, incubation at 35 °C is preferred		[22]
**QC testing of BC bottles**	Same as standard BC		[28]
**TEST RESULTS**			
**Preliminary result**	Test reports positive or negative culture results	Test reports culture positive and > 95% Gram status and morphology information	
**Final result**	Provides pathogen identification	Provides pathogens identification with resistance categories of interest (MRSA vs. MSSA, ESBL producing Enterobacteriaceae) and CRE	[29]
**Antimicrobial susceptibility testing**	Antimicrobial susceptibility determination requires separate methodologies	Antimicrobial susceptibility is included as an automated output of the test result and therapy recommendations based on local treatment guidelines	
**Data interpretation and output**	- Alert for preliminary and final report; - Capable of paper-based and electronic results to not only laboratory, but physician and ward of patient	Minimal requirements in addition to therapy recommendations based on local treatment guidelines	
**CONSUMABLES**			
**Sample collection components**	None provided	All components required for sample collection are included in the kit	
**BC bottles**	Only compatible with BC media bottles from the test manufacturer	Compatible with local manufacture of BC media bottles with a specified media formulation	
**Sample tracking/Patient identification**	Compatible with 2D barcodes and labels	Stakeholder interviews	
**Storage conditions of BC bottles**	6 months at + 5 °C to 35 °C, 70% humidity, including transport stress (48 h at 50 °C); no cold chain required	12 months at + 5 °C to + 40 °C at 90% humidity & transport stress (72 h at 50 °C); no cold chain required	High environmental temperatures and high humidity is often a problem in many countries. High environmental temperatures and high humidity is often a problem in many countries [30].
**Shipping conditions of consumables & kit**	No cold chain required; tolerance of transport stress for a minimum of 48 h at 5 °C to + 40 °C	No cold chain required; tolerance of transport stress for a minimum of 72 h at 5 °C to + 40 °C	Refrigerated transport is costly and often cannot be guaranteed during the entire transportation process. Frequent delays in transport are commonplace [30].
**Waste disposal**	Consumables should be able to be disposed of as biohazardous waste as specified by WHO guidelines according to the safe management of waste from health-care activities or per country regulations		[31]
**OPERATIONAL CHARACTERISTICS**			
**Biosafety**	Same as standard BC in a closed system; Biosafety alert is provided when a pathogen identified is on a predefined biosafety list	- No need for a biosafety cabinet; basic safety procedures need to be followed (standard PPE); - Alarms present for organisms that pose a biosafety risk for laboratory acquired infections	
**Operating conditions**	- Between + 10 °C to + 35 °C at 70% humidity and at a max altitude of 2000 meters above mean sea level; - Ability to function in a high dust environment, with manual cleaning via standard lab consumable clean wipes or cleaning tool provided with the instrument	- Between + 5 °C to + 40 °C at 90% humidity and at a max altitude of 3000 meters above mean sea level; - Ability to function in a high dust environment with minimal manual cleaning required by user	High environmental temperatures and high humidity and dust are often an issue in LMICs. High environmental temperatures and high humidity and dust are often an issue in LMICs.

MALDI-TOF MS: Matrix-assisted laser desorption/ionization-time of flight mass spectrometry; QC: Quality control; ESBL: Extended spectrum beta-lactamases; MSSA: Methicillin-sensitive *Staphylococcus aureus*; MRSA: Methicillin-resistant *Staphylococcus aureus*; CRE: carbapenem-resistant Enterobacteriaceae; WHO: World Health Organization; PPE: Personal protective equipment.

## Supplementary Materials

The following are available online at http://www.mdpi.com/2075-4418/9/1/10/s1.
