# Supplementary material for: Defining System Requirements for Simplified Blood Culture to Enable Widespread Use in Resource-Limited Settings
Source: Diagnostics (Basel). 2019 Jan 11;9(1):10. doi: 10.3390/diagnostics9010010 (PMC6468589; doi:10.3390/diagnostics9010010)
Supplement: Supplementary file 1 [file diagnostics-09-00010-s001.pdf]

Information About You

- What is your title/role?
- Background?
- Time in your field?
- Where do you work?

Purpose

- ***Enable the Development*** – of a simplified blood culture system fit for purpose to low resource areas by developing an appropriate Target Product Profile

- Support fever management to allow targeted treatment and reduce morbidity associated with sepsis.
  - What level do you think is achievable now?
  - What level would it be ideal to have blood culture available?

Health System Levels

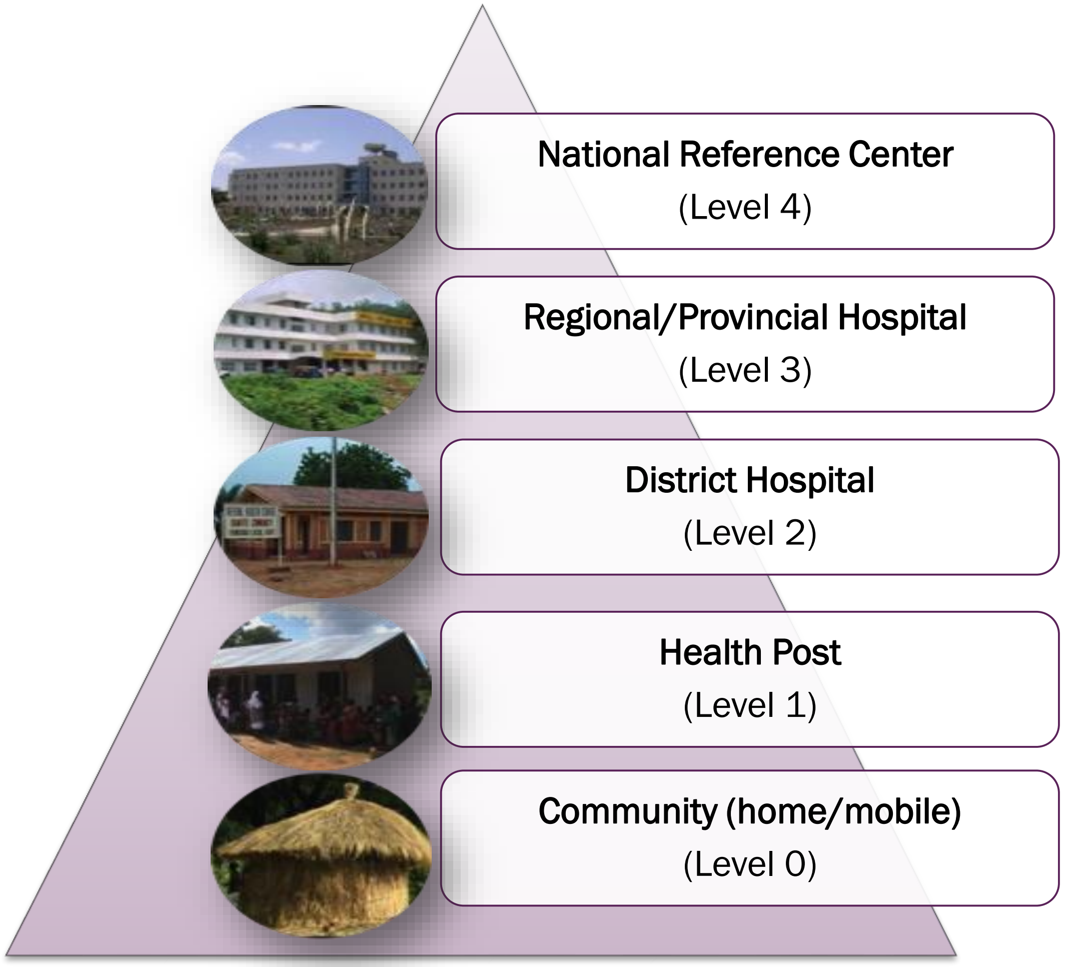

Target Product Profiles

- The purpose of a TPP is to inform product developers of key characteristics and the performance specifications of a test that are required to meet the end user's needs for a defined use case.

- A systematically developed TPP can **ensure alignment** of objectives across company departments, accelerate development timelines, minimize development risks, and eventually **lead to an optimal product** that meets user needs.
  - The TPP document is therefore an important communication tool between users and product developers

- TPPs often include an ***optimal*** and ***minimal*** definition for each test performance characteristic.
  - Ideally, products should be designed to achieve as many of the optimal characteristics that are feasible, while still satisfying the minimal criteria for all defined features.

Goal: Support fever management to allow targeted treatment and reduce morbidity associated with sepsis.

Potential case identified → **Sample collection** → **Sample transport to lab** → **Test procedure** → **Test results** → **Patient outcomes**

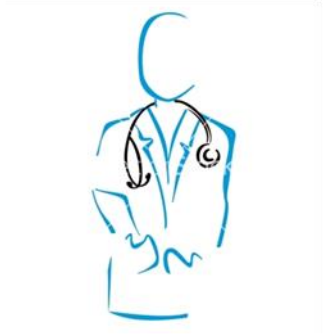

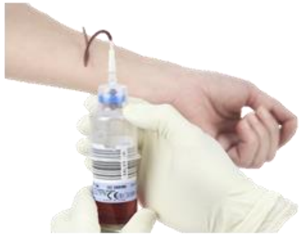

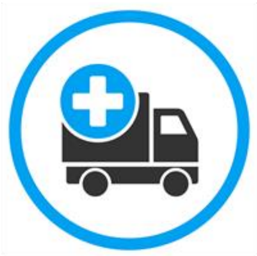

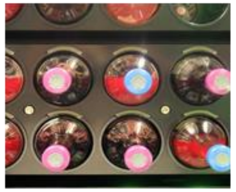

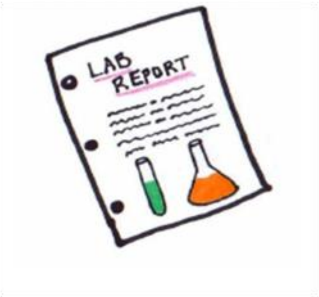

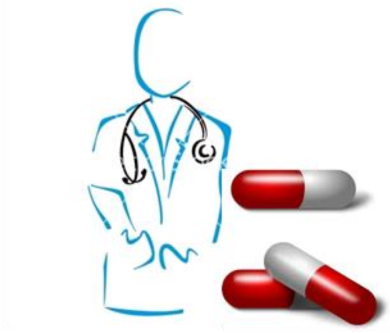

Goal: Support fever management to allow targeted treatment and reduce morbidity associated with sepsis.

Potential case identified

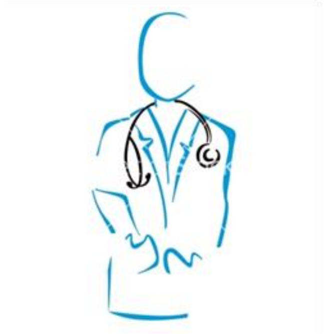

Sample collection

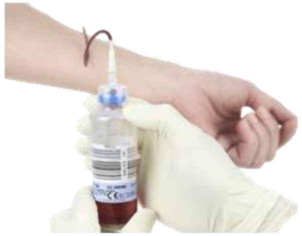

Sample transport to lab

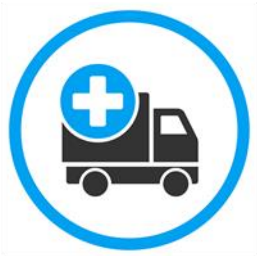

Test procedure

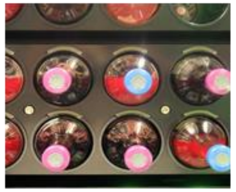

Test results

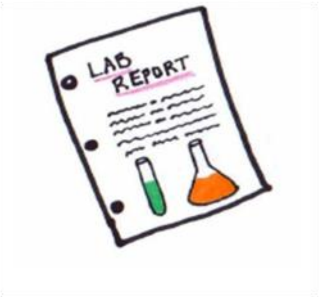

Patient outcomes

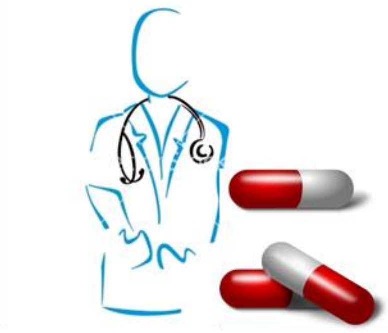

- What are the patient populations of interest (adult, pediatric, immunocompromised)?
- What level of the health system would use a simplified blood culture system and who would use it?

Goal: Support fever management to allow targeted treatment and reduce morbidity associated with sepsis.

Potential case identified → **Sample collection** → **Sample transport to lab** → **Test procedure** → **Test results** → **Patient outcomes**

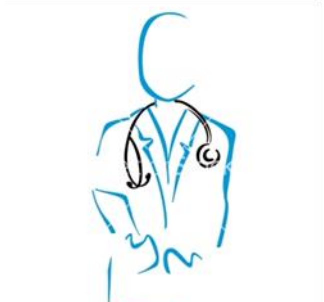

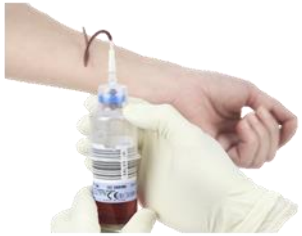

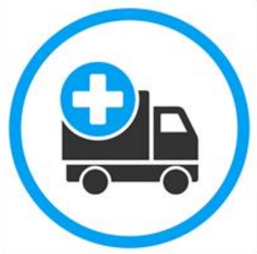

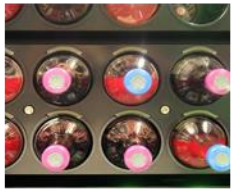

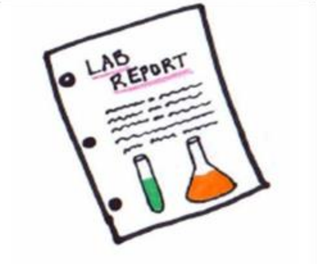

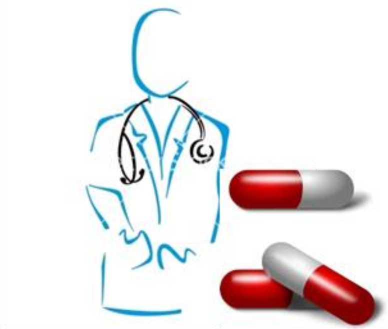

- How do you currently test for suspected bacteremia/sepsis? (Manual/Instrument, bottles, blind subculture, identification, Susceptibility testing)
- What are the challenges? What is stopping you from doing better than you are? What is stopping you from performing BC now or using it more frequently?
- What is a typical patient load/bottles per week?

Goal: Support fever management to allow targeted treatment and reduce morbidity associated with sepsis.

Potential case identified → **Sample collection** → **Sample transport to lab** → **Test procedure** → **Test results** → **Patient outcomes**

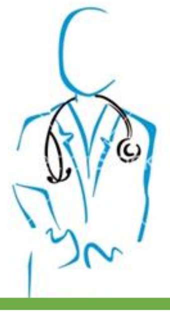

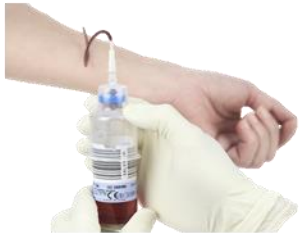

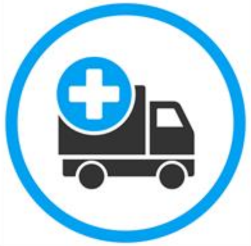

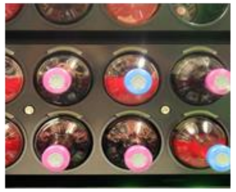

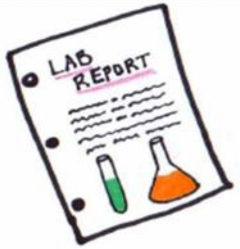

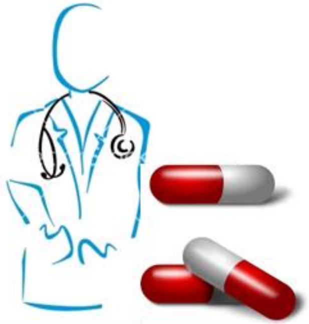

- Who would collect the sample and what would be their typical level of training?
- Blood volumes typically collected; other sterile body fluids collected?
- How is a blood culture sample typically collected? What supplies are required?

Goal: Support fever management to allow targeted treatment and reduce morbidity associated with sepsis.

Potential case identified → **Sample collection** → **Sample transport to lab** → **Test procedure** → **Test results** → **Patient outcomes**

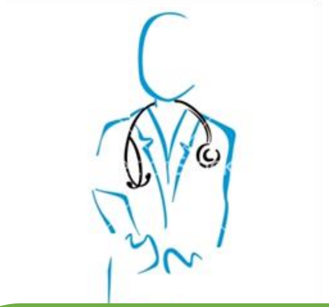

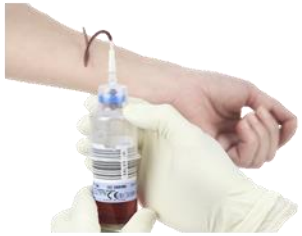

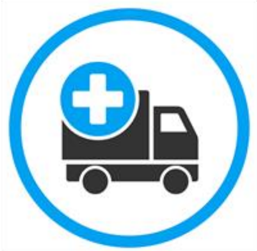

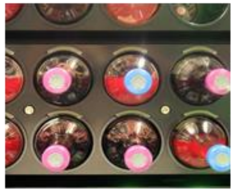

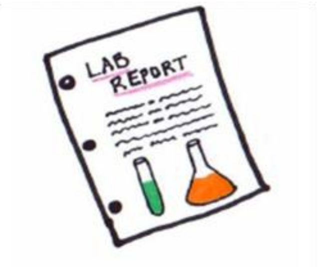

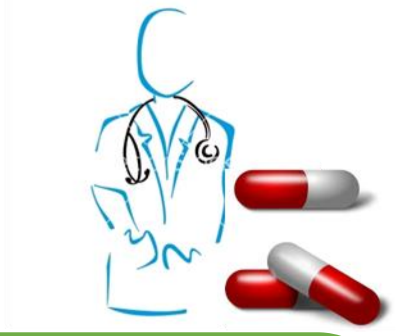

- What is the typical time between sample collection and transport to the lab?
- How is the sample stored during this time? Distance between sample collection and lab?
- How is this influenced by patient load at the clinic or the time the patient arrives at the clinic (e.g., batching?)
- How are patient ID's tracked from collection to test result?

Goal: Support fever management to allow targeted treatment and reduce morbidity associated with sepsis.

Potential case identified → **Sample collection** → **Sample transport to lab** → **Test procedure** → **Test results** → **Patient outcomes**

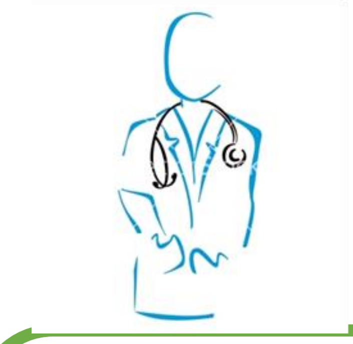

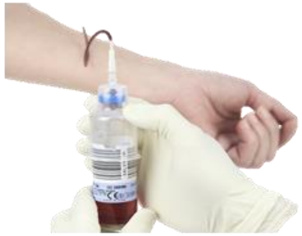

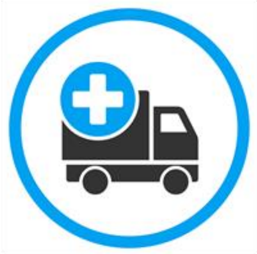

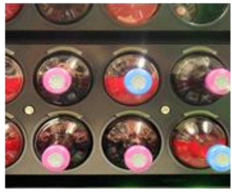

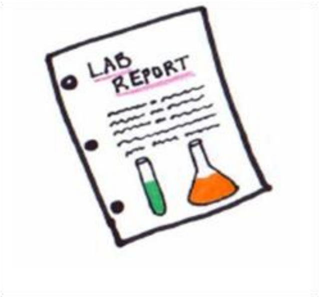

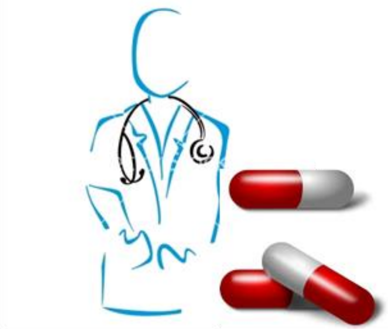

- What is the laboratory environment like where the test would be run? (e.g. Temperature, floor space, power, dust, humidity, connectivity, AC, windows open)
- What is the level of training of the staff performing blood cultures?
- What kind of quality control for blood culture do you do? How often is it done?
- How often can you order bottles and how long can they be stored (and how)? Shipping conditions / transport of supplies to the site?
- How many bottles/week? (If using instrument, what is the bottle capacity of your instrument?)
- Any critical biosafety concerns- (pos / neg bottles), value of alerts

Goal: Support fever management to allow targeted treatment and reduce morbidity associated with sepsis.

Potential case identified → **Sample collection** → **Sample transport to lab** → **Test procedure** → **Test results** → **Patient outcomes**

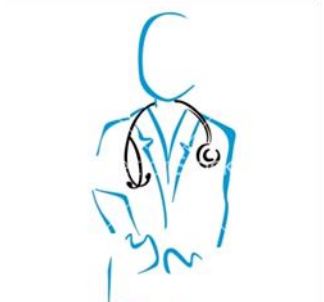

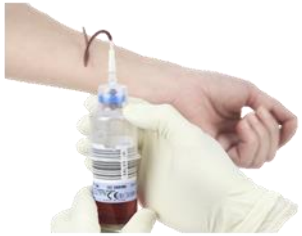

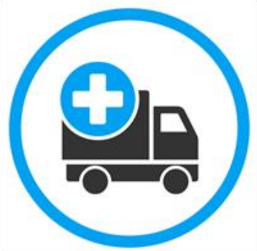

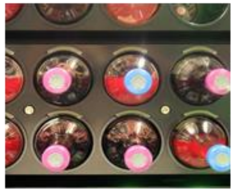

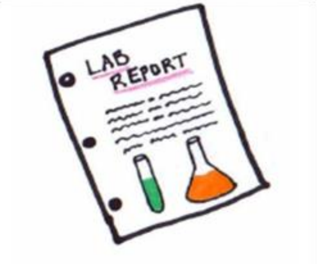

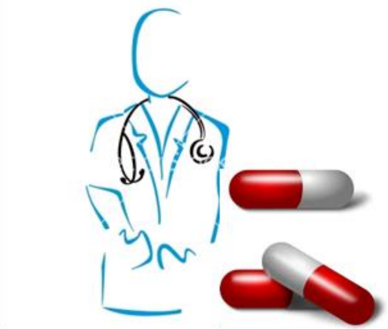

- How are results reported and to whom, timing? Are results stored? If so, how long?
- What happens if a positive result is identified? Gram stain/morphology? Identification? What method? Antimicrobial susceptibility?
- What is your percentage of contaminated blood cultures?
- What categories of pathogens would be useful for guiding treatment? E.g., GNR, Enterobacteriaceae? Etc.
- What are the most common, pathogens that you encounter? "Unusual" or regional pathogens of interest? Most important? Are anaerobes important?
- In your lab, what are the problems in terms of identification: -- e.g. Typhoid v. non-typhoidal Salmonella; Strep pneumo v. viridans or other strep, Pseudomonas v. Burkholderia?

Goal: Support fever management to allow targeted treatment and reduce morbidity associated with sepsis.

Potential case identified → **Sample collection** → **Sample transport to lab** → **Test procedure** → **Test results** → **Patient outcomes**

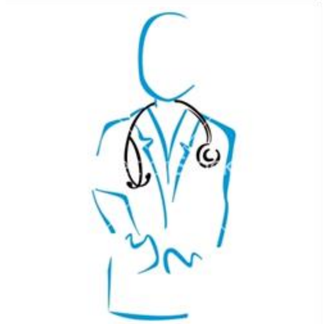

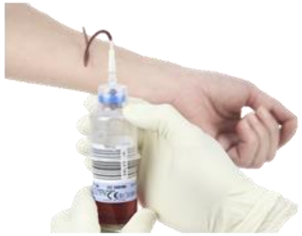

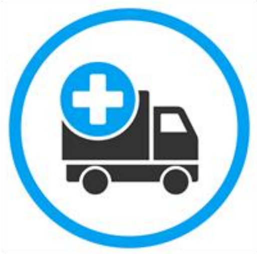

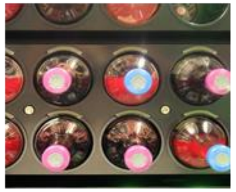

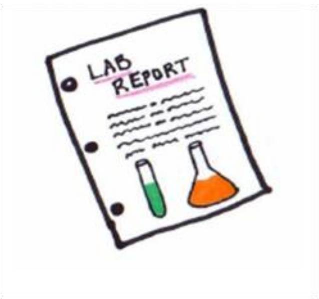

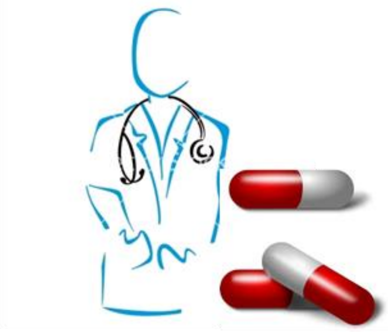

- What information do clinicians request to inform patient management?
- What treatment guidelines are used? Are the drugs available for treatment?
- What is the current turnaround time from sample collection to result? What would be ideal?
- What would be an acceptable cost?
  - (cost of bottle, cost of instrument)
- Who generally pays for such a tests (e.g. patient, MoH, funder?)

Current Pathway for Blood Culture in High Resource Countries

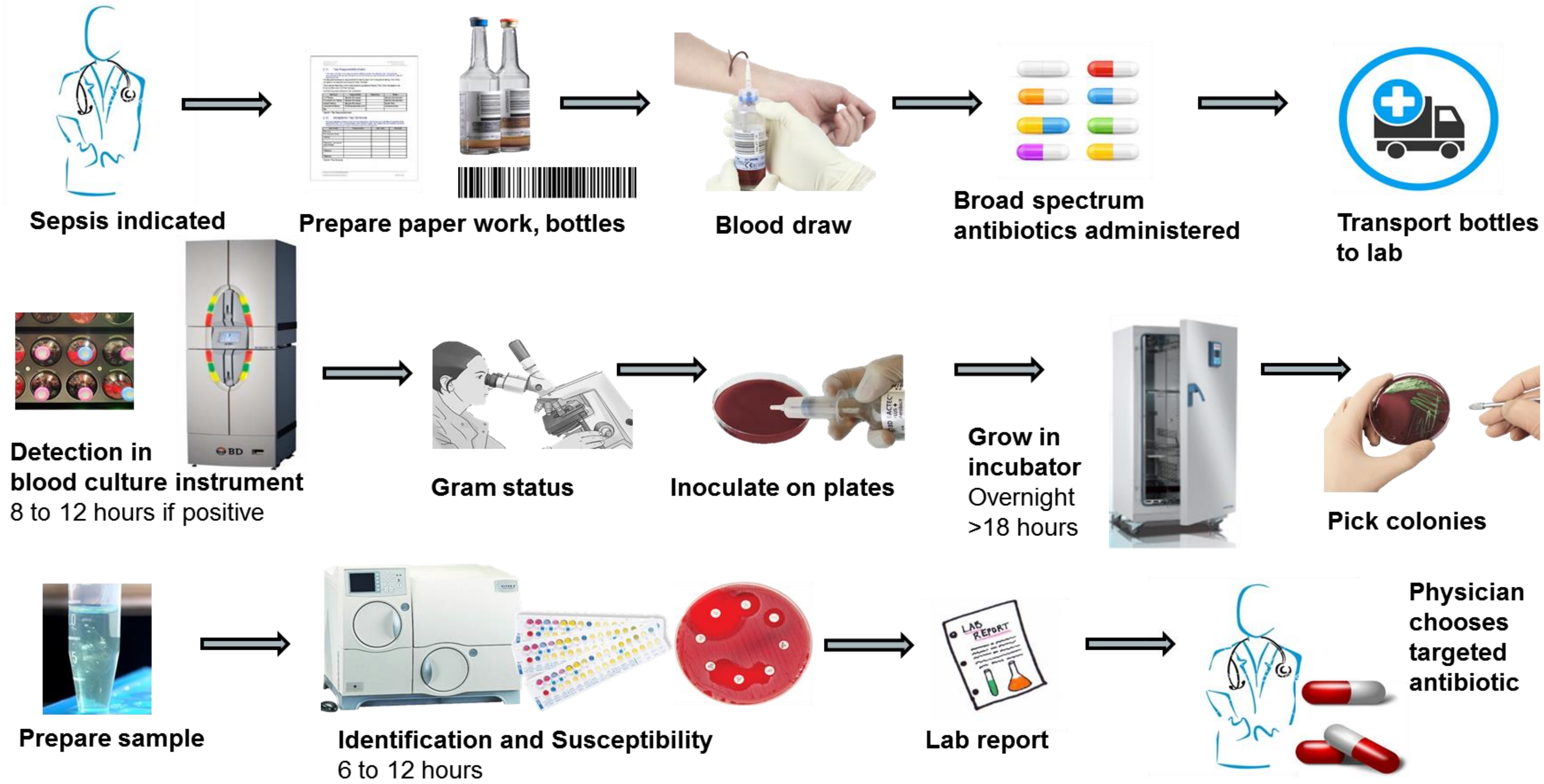

Proposed Pathway for Simplified Blood Culture

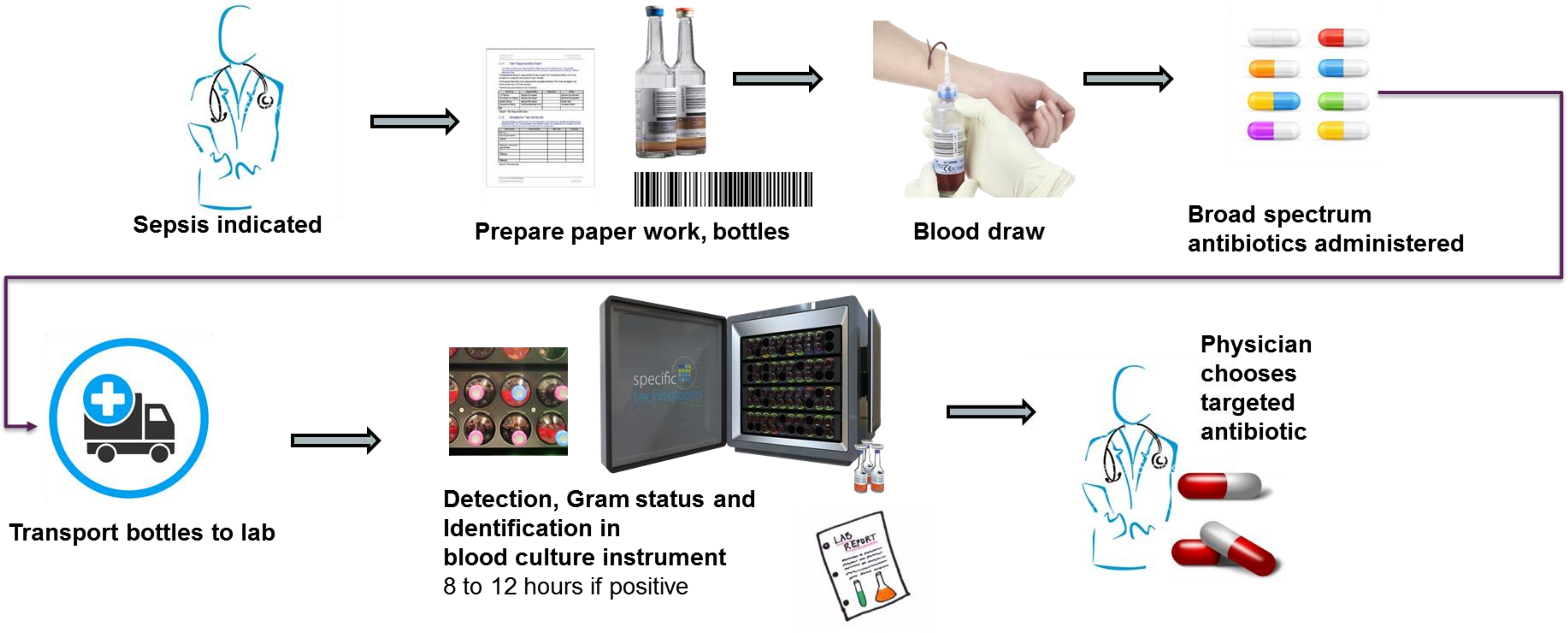

- What would the idea blood culture system look like for you?
- Would a simplified blood culture system, as described above, be helpful?
  - Why or why not?
